# The epiallelic potential of transposable elements and its evolutionary significance in plants

**DOI:** 10.1098/rstb.2020.0123

**Published:** 2021-06-07

**Authors:** Pierre Baduel, Vincent Colot

**Affiliations:** Institut de Biologie de l'Ecole Normale Supérieure (IBENS), Centre National de la Recherche Scientifique (CNRS), Institut National de la Santé et de la Recherche Médicale (INSERM), Ecole Normale Supérieure, PSL Research University, 75005 Paris, France

**Keywords:** epigenetics, transposable elements, evolution, plant genomes, adaptation, DNA methylation

## Abstract

DNA provides the fundamental framework for heritability, yet heritable trait variation need not be completely ‘hard-wired’ into the DNA sequence. In plants, the epigenetic machinery that controls transposable element (TE) activity, and which includes DNA methylation, underpins most known cases of inherited trait variants that are independent of DNA sequence changes. Here, we review our current knowledge of the extent, mechanisms and potential adaptive contribution of epiallelic variation at TE-containing alleles in this group of species. For the purpose of this review, we focus mainly on DNA methylation, as it provides an easily quantifiable readout of such variation. The picture that emerges is complex. On the one hand, pronounced differences in DNA methylation at TE sequences can either occur spontaneously or be induced experimentally *en masse* across the genome through genetic means. Many of these epivariants are stably inherited over multiple sexual generations, thus leading to transgenerational epigenetic inheritance. Functional consequences can be significant, yet they are typically of limited magnitude and although the same epivariants can be found in nature, the factors involved in their generation in this setting remain to be determined. On the other hand, moderate DNA methylation variation at TE-containing alleles can be reproducibly induced by the environment, again usually with mild effects, and most of this variation tends to be lost across generations. Based on these considerations, we argue that TE-containing alleles, rather than their inherited epiallelic variants, are the main targets of natural selection. Thus, we propose that the adaptive contribution of TE-associated epivariation, whether stable or not, lies predominantly in its capacity to modulate TE mobilization in response to the environment, hence providing hard-wired opportunities for the flexible exploration of the phenotypic space.

This article is part of the theme issue ‘How does epigenetics influence the course of evolution?’

## 1. Introduction

There is mounting evidence that heritable differences in traits can be transmitted in the absence of any DNA sequence changes. The resurgence of this concept of ‘soft-inheritance’ has led to a re-evaluation of the role of the environment in the rapid induction of heritable phenotypes independently of sequence variants [1]. In plants and mammals, variation in the epigenetic machinery, notably DNA methylation, which targets transposable element (TE) sequences to limit their mobility, appears to be an important mediator of this non-canonical system of inheritance [2,3]. Unlike mammals, plants do not extensively reprogramme DNA methylation across generations [4], thus providing a likely explanation for their apparent higher propensity to generate heritable epialleles.

TE sequences are ubiquitous components of eukaryotic genomes and they are in large part responsible for the considerable variations in genome size that can be seen even between closely related plant species [5]. Moreover, because TE sequences tend to be methylated across their entire length, they are responsible for the bulk of DNA methylation in plant genomes. This methylation affects cytosines in all possible contexts (i.e. CG, CHG and CHH, where H = A, T or C), and it is associated with other chromatin modifications, including dimethylation of lysine 9 of histone H3 (H3K9me2), in an intricate web of still partly unresolved causal chains [6]. In the reference plant *Arabidopsis thaliana*, maintenance of methylation at CG and CHG sites through replication is effected respectively by the DNA methyltransferases (DNA MTases) MET1, which recognize hemimethylated CGs at the replication fork, and CMT3, which belongs to a class of DNA MTases unique to plants that recognize nucleosomes decorated with the heterochromatic mark H3K9me2 [6,7]. Thus, whereas methylation maintenance is templated at CGs, it is based on a feed-forward loop involving histone methylation at CHGs. As for methylation at CHHs, it needs to be re-established at each replication cycle because of the asymmetric nature of these sites. Over many TE sequences, this re-establishment is carried out by so-called RNA-directed DNA methylation (RdDM), a pathway involving the production of small RNAs (sRNAs) and also responsible for de novo methylation in all three sequence contexts. However, CHH methylation at some TE sequences relies instead on another pathway involving CMT2, which acts similarly to CMT3 [6].

By contrast to TE sequences, few genes are methylated (approx. 30% of all genes in *A. thaliana*), in a pattern referred to as gene body methylation (gbM) because it is restricted to part of the transcribed region only. Furthermore, gbM affects CG sites exclusively [8]. Although genes with gbM are under selection to remain methylated, the function of gBM remains elusive [9,10].

Soon after her discovery of TEs in the 1940s–1950s, Barbara McClintock identified several TE-containing alleles in maize that caused heritable suppressible mutant phenotypes. Subsequent molecular characterization of one such allele at the *a* locus revealed that the TE insertion, which is located upstream of the *a* gene, can switch between DNA methylation states and that these epivariants can be stably inherited by themselves [11,12]. Many additional TE-containing suppressible alleles of genes have since been identified in plants using similar genetic and molecular approaches, drawing interest to epivariation as a potential source of adaptive heritable differences in traits. However, despite an extensive literature on the subject (reviewed in, e.g. [2,13–15]), there is still no consensus as to whether or not heritable epivariation plays a significant role in adaptation and evolution.

Here, we review our current understanding of DNA methylation variation at TE sequences in plants. Thanks to the development of genome or epigenome sequencing and editing techniques, our knowledge has rapidly increased over the past 20 years. While the majority of studies we discuss are in *A. thaliana*, we have also paid attention to results obtained in crops, which tend to have much larger, TE-laden genomes, as well as in non-model plants characterized notably by distinct life cycles and modes of reproduction.

After establishing a set of key definitions that should resolve lingering ambiguities (box 1), we present the different types, potential sources and functional consequences of TE-associated epivariants, before reassessing their evolutionary significance. Given the available evidence and despite possible differences among plant species, we argue that natural selection acts predominantly on the allelic variants caused by TE insertions rather than on the heritable epialleles present at some TE-containing alleles. Nonetheless, by enabling TE mobilization, TE-associated epivariation, whether stable or transient, may provide plant genomes with a powerful environmentally sensitive engine of phenotypic exploration.

Box 1. **Definitions**

**Epigenetic state:** any chromatin state, including DNA methylation, at a given locus

**Epivariation**: any variation in epigenetic state that is transmissible through cell division

**Epiallele**: an epivariant that is independent of any DNA sequence change, in opposition to an allelic epivariant

**Epimutation**: a change in epiallelic state

**Epiallelic inheritance**: the transmission of epialleles across generations. Epiallelic inheritance can be **intergenerational** if the epiallele is transmitted across one generation only (i.e. parental effects) or **transgenerational** if the transmission of the epiallele is stable across two or more generations.

## 2. TE-associated epiallelic variation

### (a) Epiallelic potential of TE-containing alleles

**Figure 1.**
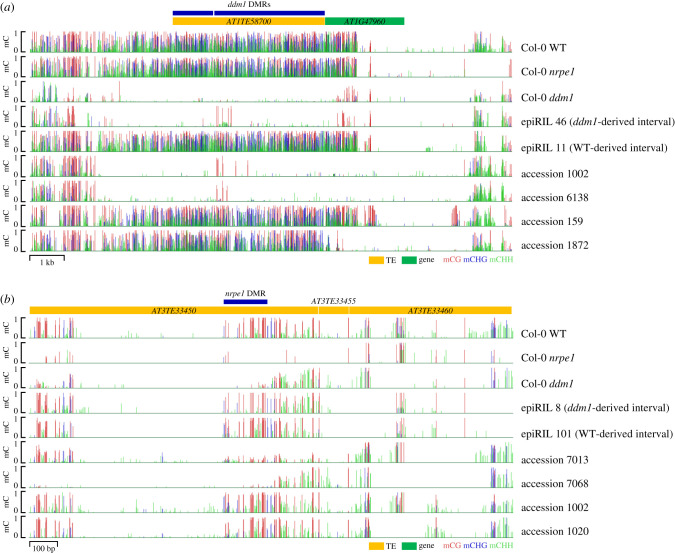
(*a*) Example of *ddm1*-induced TE-associated epivariation stably transmitted (i.e. epiallele) through at least eight generations of selfing in the epiRILs and also found in nature (*b*). Example of *nrpe1*-induced TE-associated epivariation transmitted through at least one generation with wild-type (WT) RdDM [22] and also found in nature as well as in *ddm1*, where it overlaps a reverting epivariant. *NRPE1* encodes the largest subunit of RNA Pol-V, essential to RdDM [23]. mC: level of methylation (0–100%) of each cytosine along the two genomic regions shown. Sequence coverage (not shown) was used to verify that all accessions carry the reference TE sequence at the loci of the differentially methylated regions (DMRs). (BS-seq obtained for natural accessions from the 1001 Genomes project [24], for *nrpe1* from Wendte *et al*. [25] and for epiRILs as well as WT and *ddm1* parents from G. Bohl-Viallefond, L. De Oliveira, P. Baduel, V. Colot 2021, unpublished data).

The development of genomic and epigenomic methodologies over the past 20 years has enabled the massively parallel assessment of the epiallelic potential of TE-containing alleles in plant genomes. The most complete studies to date were performed in *A. thaliana*, using mutant lines defective in either *MET1* or *DDM1*, which encodes a chromatin remodeller that is thought to facilitate access of DNA MTases to TEs as well as other repeat sequences and when mutated leads to a loss of methylation in the three contexts [16–18]. The epiallelic nature and inheritance of the strong hypomethylation induced mostly at CGs by *met1* or at all Cs by *ddm1* over TE-containing alleles was evaluated by first crossing the mutant parent to an isogenic wild-type parent. F2 individuals without the *met1* or *ddm1* mutations were then used to propagate so-called epigenetic recombinant inbred lines (epiRILs) through single-seed descent [19,20]. Thus, the epiRILs enable genome-wide, population-level surveys of the transgenerational stability of TE-associated epialleles, i.e. of the epivariants that are independent of the genetic trigger used to create them in the first place (see definitions in box 1). Results obtained with the *met1*-derived epiRILs turned out to be difficult to interpret because of the appearance of numerous non-parental DNA methylation variants in the F1 and subsequent generations [19,21] and also because of a high rate of lethality (30%) among lines [19]. By contrast, very few *ddm1*-derived epiRILs were lost during their propagation [20] and non-parental DNA methylation variants are rare in these lines, thus facilitating the analysis of inheritance patterns of parental differences. Results indicated that approximately one-third of TE sequences that lost DNA methylation in the *ddm1* parental line were inherited from that parent in the hypomethylated state across at least eight (and presumably many more) generations, thus revealing a large potential for bona fide heritable epiallelic variation in *A. thaliana* (figure 1*a*). The other two-thirds of parentally hypomethylated TE sequences regained wild-type methylation progressively, within three to five generations [26], and in either some or all of the epiRILs that contain the corresponding *ddm1*-derived chromosome intervals (figure 1*b*) [20,27]. This comprehensive survey thus revealed that epivariants at TE-containing alleles differ greatly in their properties, from a substantial proportion bearing the potential for epiallelic inheritance to many being incapable of stable transmission independently of their trigger.

This differential potential for epiallelic inheritance was found to result in large part from variations in RdDM targeting efficiency. Indeed, reversion to wild-type DNA methylation positively correlates with the abundance of matching sRNAs involved in RdDM and it is compromised in RdDM mutant backgrounds [26]. A similar correlation is also observed for hypomethylated TE-containing alleles that were generated using a partial loss-of-function *met1* mutant parent [28]. However, additional mechanisms, including histone deacetylation, are also involved in the reversion to the methylated state at a subset of RdDM targets [29] and most targets can in fact recover DNA methylation when this pathway is compromised for just one generation [22]. These reverting TE-associated epivariants are preferentially found within the pericentromeric, TE-rich regions of chromosomes and are characterized by relatively high levels of residual CG and CHG methylation, even when the sRNA-producing arm (Pol-IV) of the RdDM pathway is defective [22]. Consistent with this last observation, targeted DNA methylation through the second arm of RdDM (Pol-V) can occur with a level of independence from sRNA production [30].

By contrast, a small number of RdDM targets located preferentially within the gene-rich chromosome arms fail to restore DNA methylation in wild-type progeny of RdDM mutants. These targets gain in the mutants active euchromatic marks [22] that recruit the DNA demethylase ROS1, thus preventing remethylation upon restoration of RdDM [31]. Indeed, loss of ROS1 activity is sufficient to enable reversion to the methylated state at most such RdDM targets [22]. Conversely, forced expression of *ROS1* when RdDM is compromised leads to the generation of stably inherited epialleles at many of the reverting RdDM targets located on chromosome arms [32]. Furthermore, stable inheritance of hypomethylation can also be forced over some reverting RdDM targets when these are fully demethylated using a CRISPR-dCas9–TET1-targeted demethylation system [22]. Together, these observations suggest that the complete loss of DNA methylation at RdDM targets abolishes all possibility of reversion to the methylated state. In other words, stable epiallelic inheritance seems to rely on total erasure of DNA methylation. Incidentally, this conclusion challenges the notion of RdDM as a de novo DNA methylation pathway. However, RdDM was defined as such using mainly transgenes that provide an artificial supply of sRNAs in large amounts to direct the methylation of target sequences *in trans* [33,34]. Thus, the fact that some RdDM targets can lose DNA methylation irreversibly would indicate either that they are single copy or that related TE sequences elsewhere in the genome are not a sufficient source of sRNAs to enable remethylation *in trans*, such as is seen in the extreme case of paramutation [35,36]. An illustration of this point can also be found in the *ddm1*-derived epiRILs at the *FWA* locus, an RdDM target that contains an ancestral, highly degenerate TE sequence with no match elsewhere in the genome [37]. This locus only suffers a moderate loss of DNA methylation in the parental, early generation *ddm1* line, which is robustly reversed in the epiRILs. However, a handful of lines harbour instead a fully demethylated, stably inherited epiallele [20], which mirrors the sporadic occurrence of this epiallele in advanced *ddm1* generations [38,39]. Similarly, the partial loss-of-function *met1* mutant generates stably inherited hypomethylation variants, but only over TEs where methylation loss is complete not only at CGs, but also at CHGs and CHHs [28].

Restoration of DNA methylation can also take place through pathways independent of RdDM, but only over TE genes, which are found within 15% of all annotated TEs. Unlike the non-coding TE sequences that flank them, TE genes are typically not targeted by RdDM [40] but their CHH as well as CHG methylation is immediately recovered in the progeny of complementation crosses between genetically unlinked mutants affected in the CMT2- and CMT3-dependent pathways [40]. By contrast to what is observed in *ddm1*, CG methylation remains largely unaffected in these mutants and appears to be necessary for the recovery of CHH and CHG methylation in the complemented progeny. Indeed, as with some RdDM targets [22], a small number of CMT2/3 targets fail to regain methylation upon complementation and this stable hypomethylation is associated with a loss of CG methylation near the extremities of TE genes. Moreover, like at non-reverting RdDM targets, stable hypomethylation is associated with the loss of the heterochromatic mark H3K9me2. Although the exact mechanisms of this loss remain to be determined for RdDM targets, they involve for TE genes the histone demethylase IBM1, which acts over transcribed protein coding genes to prevent indirectly their methylation at CHG sites [41–43]. Together, these findings point to a role for leftover CG methylation and other associated chromatin marks as a memory system for directing remethylation after accidental loss at both RdDM and non-RdDM targets, thus preventing the stable inheritance of the hypomethylated state [22,40].

Results presented so far imply that differences in genetic backgrounds caused by variations in TE copy-number could also have a substantial impact on the transgenerational epiallelic stability of a given TE-containing allele. Indeed, there is a near universal positive correlation between the size of a TE family and the strength of epigenetic silencing of its members [44–46]. This correlation is also in line with the copy-number-dependent de novo DNA methylation of new insertions observed for *ATCOPIA93*, a particularly active TE family in *A. thaliana* [47,48]. Conversely, TE sequences that are demethylated in partial loss-of-function *met1* mutants are less prone to regaining methylation if they are in few rather than in many copies [28]. Given that copy-number for most TE families varies extensively among *A. thaliana* accessions [48,49], we can, therefore, expect the epiallelic potential of the very same TE-containing allele also to differ between genetic backgrounds. Thus, replicating in a panel of non-reference accessions and extending to species with larger genomes the genetic studies described above will likely bring invaluable information for our understanding of the epiallelic potential of TE sequences in plants.

### (b) TE-associated epivariation in nature

**Figure 2.**
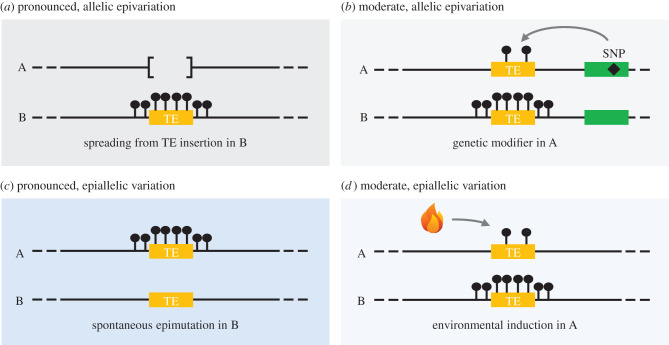
The two different types (allelic versus epiallelic) and flavours (pronounced versus moderate) of TE-associated epivariation and their possible sources in nature. SNP, single nucleotide polymorphism.

While genetic studies are powerful tools, they cannot inform us as to what extent the potential for epiallelic variation at TE sequences unfolds in nature. Thanks mainly to large scale efforts that have culminated in the determination of the DNA methylome of hundreds of *A. thaliana* accessions taken from across the world, quantitative answers to this question are emerging. Indeed, results of these DNA methylome analyses revealed extensive epivariation at the regional level between accessions, in large part over TE sequences [24,50,51]. However, as illustrated in figure 2 and discussed in the next sections, establishing the allelic or epiallelic nature of the myriad of TE-associated epivariants thus uncovered and identifying the genetic, spontaneous or environmental factors involved in their generation and stability in nature remain challenging.

Also, it should be pointed out that because methylome data were obtained using plants propagated for a few generations in the laboratory rather than directly collected from the wild, an indeterminate number of epivariants may originate from seed bulking. More importantly, this propagation step should lead to the under- and over-reporting of fast-reverting and stable natural epivariants, respectively. Perhaps as a consequence of this inherent ascertainment bias, the majority of differentially methylated regions between accessions are associated with DNA sequence changes *in cis* [50,51]. Similar results were reported for maize based on comparisons of methylome data for several populations of modern maize and landraces [52]. More specifically, most gain of DNA methylation epivariants are low frequency and tend to be associated with the presence of rare non-reference TE sequences or the absence of reference TE sequences (figure 2*a*) [49,50,52], a type of sequence polymorphism that is abundant among *A. thaliana* accessions and maize lines [48,49,53,54]. In *A. thaliana*, these *cis* associations with TE insertion polymorphisms are likely causal given that the vast majority of TE sequences are methylated in any given genome and that DNA methylation can spread over several hundred base pairs into surrounding regions [48,55]. Spreading was also observed in maize and rice [56–58], thus establishing the generality across plant species of the impact of TE sequences on the methylation status of adjacent regions. In turn, these findings highlight the need to take into account TE insertion polymorphisms before concluding that heritable epivariants are true epialleles. Moreover, investigations in maize of sites where DNA transposons have been inserted then excised show little evidence of an epigenetic memory [58]. Thus, even when excision restores precisely the original target site, it is unlikely to provide an efficient means by which heritable bona fide epiallelic variation can be generated. In the light of this consideration, it is in turn unclear if the DNA methylation observed in *A. thaliana* in regions adjacent to deletions [49] reflects true epiallelic inheritance following clean excision rather than allelic epivariation maintained because of the excision footprints that most DNA transposons leave.

The large amount of methylome data obtained from natural *A. thaliana* accessions was leveraged to perform genome-wide association studies (GWASs), which identified major *trans* modifiers of DNA methylation variation at TE sequences (figure 2*b*) [51,59]. Three *trans* modifiers stand out as they map to genes known to be involved in RdDM (*NRPE1*, *AGO1*) or other DNA methylation pathways that target TEs (*CMT2*) [60]. However, the range of DNA methylation differences associated with natural variation at these genes is more limited compared with that achieved using experimental knockout (KO) mutants [59]. In fact, the reduction of mCHH explained by the derived alleles of *NRPE1* at RdDM-targeted TE sequences resembles that of experimentally generated hypomorphic alleles with similar sequence defects [54]. In turn, these observations suggest that severe and widespread loss of methylation at TE sequences is strongly counter-selected in nature, a conclusion further supported by the fact that the derived alleles of *CMT2* and *NRPE1* associated with reduced mCHH are rarely present together in nature and much less so than expected by chance [59]. Conversely, despite the apparent lack of overt phenotypic consequences of moderate loss of mCHH over TE sequences reported so far, accessions carrying the derived alleles of *CMT2* and *NRPE1* are not distributed randomly across the world but rather in relation to specific climates [24,51,59,61]. Thus, it is tempting to speculate that the moderate reduction of mCHH caused by genetic *trans* modifiers over thousands of TE sequences across the genome participates in local adaptation.

Although these genetic *trans* modifiers provide a natural counterpart to those used experimentally to determine the epiallelic potential of TE-containing alleles, it is not known what fraction of the natural epivariants they generate represent true epialleles. As a matter of fact, based on the many lines of experimental evidence indicating that residual methylation prevents stable inheritance of the hypomethylated state, the moderate loss of mCHH caused by the derived alleles of *NRPE1* and *CMT2* is unlikely to generate true epiallelic variation. In marked contrast, a sizable fraction (between 30 and 40%) of the stable TE-associated epialleles identified experimentally using *ddm1* or null RdDM mutants overlap with epivariants of similar ‘flavour’ (i.e. pronounced hypomethylation at CG, CHG and CHH sites) in nature [22,62] (figure 1). We can, therefore, assume that these epivariants are also stably inherited independently of any DNA sequence change *in cis* or *in trans*, thus representing bona fide natural epialleles. However, the natural counterparts of *ddm1*- or *nrpd1*-induced epialleles are presumably not generated through genetic deficiencies that cause strong and widespread DNA methylation loss, because such deficiencies become rapidly non-viable upon repeated selfing (e.g. [63,64]). Indeed, *ddm1* deficiencies or null *nrpd1* alleles have not been observed in nature. Thus, we must conclude that natural stable epialleles are most likely generated either spontaneously or in response to the environment.

### (c) Spontaneous generation of heritable TE-associated epialleles

**Figure 3.**
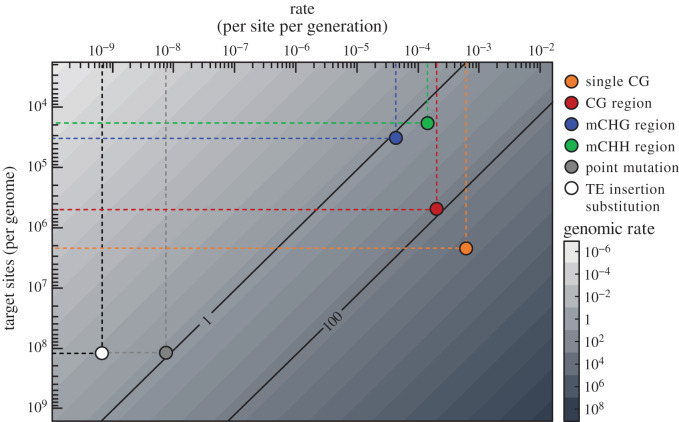
Rate of occurrence per genome for point mutations, TE insertions, point epimutations at CGs, and region-level epimutations at CGs, CHGs and CHHs (data obtained from Denkena *et al*. [69] and Baduel *et al*. [54] for TE insertion substitution rates). Genomic rates were obtained from measures per site per generation multiplied by the target size per genome. For CHG and CHH region-level epimutations we only report the rates of methylation loss, as for most TEs the methylated state is prevalent. Genomic rates of 1 and 100 events per generation are indicated by the two diagonal lines.

Methylomes have been obtained for a number of mutation accumulation lines in *A. thaliana*, thus enabling the determination of the rate at which spontaneous heritable epimutations occur [65]. Results revealed an extraordinarily high rate of gain or loss of methylation at CGs, which is about five orders of magnitude greater than that of point mutations [66–68] (single CG, figure 3). However, most of these epimutations at CGs occur in isolation and are, therefore, likely inconsequential, given the paucity of known examples of single C epivariants with a functional impact. Concerted gain or loss of epimutations at consecutive CG sites are nonetheless relatively and equally frequent (CG regions, figure 3). They affect genes with gbM mainly [66,67,69], again with no obvious functional consequences [70]. A third class of spontaneous epimutations affect TE sequences predominantly and result in most cases in a loss rather than a gain of methylation, and in all three contexts [67] (figure 2*c*). These epimutations, therefore, resemble the stable epialleles induced experimentally using *ddm1* or null *nrpd1* mutants (figure 1) and indeed it was shown that over half of these epimutations are transmitted across generations [71]. Furthermore, they occur at rates per methylated region (mCHG and mCHH regions; figure 3) that are orders of magnitude higher than the rate of mutations per nucleotide [69]. However, because TE sequences occupy only 20% of the genomic space in *A. thaliana*, the spontaneous epimutation rate per genome is in fact very similar to that of point mutations [69,71] and only an order of magnitude above the TE insertion substitution rate measured in nature (figure 3) [54].

We have already mentioned that the moderate loss of DNA methylation induced by *ddm1* at the RdDM target *FWA* can translate sporadically into a complete loss in subsequent generations [20,38,39]. Thus, it is reasonable to assume that the spontaneous rate of TE-associated epimutations may be higher in accessions with lower mCHH over TE sequences, such as those carrying derived alleles of *CMT2* and *NRPE1* [59]. Consistent with this idea, non-mobile TE families are less methylated than mobile ones, presumably because of weaker targeting by RdDM, and indeed more prone to epimutations [48]. In turn, this observation suggests that spontaneous epimutations occur predominantly over ancestral and therefore widely shared TE-containing alleles. Determining the generality of this conclusion is key as it could have major implications regarding the differences of epiallelic potentials between accessions. Finally, we know that some active TEs have the ability to *trans*-demethylate other members of the same TE family [72,73]. This finding implies that the reactivation of one TE copy through spontaneous epimutation could ultimately impact many other TE copies belonging to the same family, in effect multiplying the spontaneous epimutation rate across the genome.

Thus, evaluating to what extent genetic backgrounds, in terms of both genetic modifiers and TE landscapes, influence the rate of spontaneous epimutations is crucial for our understanding of their evolutionary significance.

### (d) Environmentally induced TE-associated epimutations

In addition to being generated spontaneously, epimutations could be induced by exposure to environmental stresses (figure 2*d*). Indeed, changes in epigenetic states, often affecting TE sequences, have been described in response to environmental stresses, whether biotic or abiotic, in *A. thaliana*, crops (maize, rice, wheat, barley etc.) and trees (*Populus* and *Quercus*) (e.g. [74–76]). Detailed studies in *A. thaliana* indicate that most changes in DNA methylation induced by salt or drought stress or by mild temperature variations reside within TE sequences (respectively [51,77,78]). Furthermore, the epialleles induced by one stress show little overlap with those induced by another [78], which suggests a significant degree of specificity in the epigenetic responses of TEs to any given stress. Given that the epiallelic variation identified in these experiments tends to be restricted to CHGs and CHHs [77,78], we can expect it to be less stably inherited than that resulting from spontaneous epimutations that affect all C contexts. Indeed, even though a significant fraction of environmentally induced epialleles are transmitted to the next generation in *A. thaliana* [75,79–83], rice [84] and maize [85], transmission of DNA methylation changes across two or more generations is rarely observed [77–79,85–88]. As a matter of fact, when stress is applied during the reproductive phase [75,79,81], gametes leading to the first generation offspring are also exposed and parental effects, therefore, cannot be ruled out.

In a few notable cases, environmentally induced epialleles in *A. thaliana* are transmitted further than one unexposed generation. Such transgenerational epiallelic inheritance was observed following drought [87], salt [77], UV as well as heat and cold stress [89]. However, these heritable epialleles are only a minute fraction of all the epialleles induced by stress. Furthermore, many are lost within a few generations [77] and they are almost never shared between stress-exposed lineages [87], which suggests that they occur stochastically at high rates under these conditions. Such a role of the environment in modulating spontaneous epimutation rates, which must be confirmed experimentally, would have important evolutionary implications, as discussed below.

Finally, it was shown that environmentally induced intergenerational epiallelic inheritance is significantly increased when stress exposure is repeated over multiple generations [77,90], indicating that they could be less transient in perennial plants, thanks to their longer life cycles.

### (e) Additional determinants of TE-associated epiallelic variation?

The study of non-model plants will likely reveal additional determinants of TE-mediated epimutations. Indeed, many plants, including a number of crop species, propagate vegetatively or asexually and it is now well established that artificially induced regeneration from vegetative tissues leads to the appearance of epimutations that are at least partially heritable in rice [91], maize [92] and oil palm [93] as well as in *A. thaliana* [94,95]. In oil palm, micropropagation through cell culture of leaf primordia followed by plant regeneration was shown to be associated with severe loss of methylation at thousands of TE loci and, in at least one case, this loss can be transmitted to the progeny [93]. In *A. thaliana,* plants regenerated from root tissues, which compared with leaf tissues exhibit moderate hypomethylation at a small number of sequences including TEs [96], tend to transmit the majority of these hypomethylated regions in a more severely hypomethylated form, for at least three generations [95].

The mechanisms by which an initially moderately hypomethylated epiallele can turn during asexual reproduction into a heritable, strongly hypomethylated epiallele that resembles *ddm1*-induced epimutations [95] are not known. However, several lines of evidence suggest that this transition relies on differences in DNA methylation dynamics between asexual and sexual modes of reproduction. Indeed, CHH methylation is re-established through strong RdDM activity during sexual reproduction in *A. thaliana* [4,97–99]. By contrast, RdDM activity appears comparatively weak in *A. thaliana* cell cultures, as indicated by the depletion of mCHH and the loss of 24 nt siRNAs observed during vegetative propagation in *A. thaliana* [94,100] as well as during *in vitro* propagation of oil palms [93]. Although it is not known if RdDM activity is also reduced during naturally occuring asexual reproduction, were this the case, some environmentally induced hypomethylation could gain transgenerational stability in this context (a hypothesis also explored by Mounger *et al.* [101] in this theme issue). Supporting this prediction, stress-induced epiallelic variants are transmitted to the next generation in triploid dandelions [102], which typically reproduce asexually through apomixis [103]. Moreover, the occurrence of these epiallelic variants is accompanied by a global reduction of RdDM-associated sRNAs that persists at least across two unstressed generations [104]. Finally, a comparison of DNA methylomes across diverse angiosperms identified a trend for lower levels of mCHH in species with histories of clonal propagation [105]. Given that sexual reproduction is facultative in numerous plants that can reproduce instead through apomixis or vegetative propagation [106], stable environmentally induced epiallelic variation may be more prevalent in the plant kingdom than indicated by studies in *A. thaliana* or other model plants with obligate sex.

## 3. Adaptive potential and evolutionary significance of TE-associated epiallelic variation

### (a) Functional consequences of stable TE-associated epiallelic variation

Stable TE-associated epialleles, whether induced by vegetative propagation or using mutants deficient in DNA methylation, have been associated with various phenotypic consequences, from sterility in oil palm [93], to heritable variation in complex traits such as flowering time, root length and responses to biotic stresses in the *A. thaliana ddm1*-derived epiRILs [22,62,95,107]. However, the amplitude of the quantitative phenotypic differences observed in the epiRILs is at most a quarter of that observed for the same traits between accessions [108], with few exceptions. One extreme case is the extensive delay in flowering caused by the complete loss of methylation at *FWA* in a few lines [20], similar to that seen sporadically in advanced *ddm1* generations [38,39]. Remarkably, severely hypomethylated *FWA* epialleles have not been observed in nature, presumably because of the dire consequences they would have on reproductive success in this setting [109]. Furthermore, because the quantitative trait loci (QTL^epi^) identified in the epiRILs span hundreds of TE-containing alleles with stable epiallelic inheritance [62], we should bear in mind the possibility that it is the concerted epivariation across all of these alleles at any given QTL^epi^ that is causal. In this case, given that genome-wide hypomethylation like that induced by *ddm1* has not been observed in nature, the few and genomically dispersed natural counterparts of *ddm1*-induced stable epiallelic variants present in any accession would be unlikely to have any appreciable phenotypic impact, except in rare cases. These considerations may force us to revisit the notion that TE-associated epiallelic variants can jump-start heritable variation in the absence of standing DNA sequence variation [110].

In addition, all available evidence suggests that in sexually reproducing plants, stable epiallelic variants arise spontaneously in nature rather than because of severe genetic deficiencies or in response to the environment. In *A. thaliana*, such spontaneous epimutations appear mainly in the form of variations in gbM, for which a functional role is lacking [70,111]. Although some TE-associated stable epialleles have detectable phenotypic impact, the spontaneous rate of appearance of this type of variants is similar to that of single nucleotide polymorphisms (SNPs) [69]. Barring the possibility that such low epimutation rates are a property of the genetic background or the controlled environments used in mutation accumulation studies, we must conclude that spontaneous epimutations can only contribute minimally to rapid adaptation [112,113].

Nonetheless, because of the multitude of TE presence/absence polymorphisms that typically segregate within species, epiallelic potentials may differ substantially between populations. A first indication that this is the case is provided in *A. thaliana* by the example of stable natural epiallelic variation at a TE-containing allele of the gene *PPH*, which results in marked differences in leaf senescence between strains containing the TE insertion, a type of variation obviously not available in strains devoid of it [114]. Furthermore, stable TE-associated epialleles are mostly located within the gene-rich chromosome arms in *A. thaliana*, which are constantly hit by TE insertions [48,49,54]. Although the resulting TE-containing alleles remain generally at low frequency, collectively they are abundant across the species. Thus, we can expect a multiplicity of situations similar to that observed at *PPH*, each specific to a small number of *A. thaliana* accessions.

Another, broader impact of TE epivariation is of course on TE mobilization and therefore on the capacity of genomes to generate new TE-containing alleles. This is well illustrated in the *met1-* and *ddm1-*derived epiRILs where transposition is triggered for a number of TEs [19,20,115], and the resulting TE-containing alleles tend to have major effects on nearby genes, because or independently of their epiallelic properties [115]. In nature, similar large-effect alleles are constantly generated and, because most are strongly deleterious, they are rapidly purged by purifying selection, thus resulting in a fast turnover of TE landscapes [45,51]. Therefore, the emerging picture is one where the phenotypic space explored through stable epiallelic variation alone is much narrower than that probed by TE mobilization. However, because epivariation, whether stable or not, can modulate both transposition and the functional consequences of new insertions, it likely plays a major role in the adaptive potential of genomes. Ultimately though, natural selection should have much less evolutionary significance at the epiallelic than allelic level.

### (b) Functional consequences of environmentally induced TE-associated epiallelic variation

TE-associated epialleles that are experimentally induced in *A. thaliana* by compromising RdDM or through somatic embryogenesis show a strong enrichment at loci involved in defence against pathogens [22,95]. These observations indicate that TE-containing alleles with epiallelic potential could be selected positively at immune response genes. Supporting this hypothesis, mutations in pathways involved in DNA methylation or demethylation of TEs affect resistance to pathogens [81,116–119]. Moreover, upregulation of defence genes relies in many cases on active DNA demethylation of TE sequences located in their promoters [120,121]. Specifically, it was shown that ROS1 antagonizes the action of RdDM over transcription factor binding sites that are adjacent to TE sequences within the promoters of defence genes, thus exacerbating their induction in response to pathogen attacks [121]. In addition, *ROS1* expression itself is quantitatively and positively coupled to the DNA methylation level of a TE sequence located in the promoter of the gene, which as a result serves as an epigenetic rheostat or ‘methylstat’ [122,123]. Given that DNA methylation levels at TE sequences can be modulated by temperature but not uniformly across the genome [51], we can expect environmental cues to impact in complex ways the regulation of ROS1 targets, with potentially important consequences for disease susceptibility.

TE-associated epivariants induced by abiotic stresses are also often located near genes involved in the response to these insults [77,88,124–128], suggesting a regulatory role [129], but causality was demonstrated in only one case [126]. We should emphasize, however, that not all abiotic stresses induce epivariation at responsive genes, as illustrated by the lack of any direct link between TE-associated epiallelic variation and gene expression changes in *A. thaliana* plants subjected to mild drought [78,87].

Some environmentally induced epivariations are likely involved in intergenerational stress memory, which enables the second generation to outperform the first when exposed to the same stress. Indeed, *A. thaliana* mutants defective in sRNA production do not exhibit the inherited resistance to herbivory of wild-type plants [82]. Moreover, offspring of plants exposed to salt stress are pre-adapted but this adaptive response is lost when RdDM or active DNA demethylation pathways are impaired [77].

However, mechanisms exist that prevent the transgenerational inheritance of stress-induced TE-associated epiallelic variations. In *A. thaliana*, inheritance of heat-stress-induced transcriptional reactivation of TE sequences is observed in the progeny of *ddm1 mom1* double mutants, but not in the progeny of the single mutants [130]. Even though the molecular mechanism of transcriptional silencing by MOM1 does not involve DNA methylation [131], this last observation indicates that two pathways are acting redundantly in *A. thaliana* to prevent the inheritance of stress-induced epigenetic changes. Thus, at least in organisms with similar life history to *A. thaliana*, long-term epiallelic heritability of environmental changes may be selected against.

As already mentioned, even when transient, epiallelic variation at TEs may favour their mobilization. Most studies so far have only documented transcriptional reactivation of TEs in response to stress (see reviews [129,132–134]), but evidence is accruing that links this reactivation to mobilization. A role for RdDM in preventing TE mobilization in response to stress has been reported in maize [135] and is also well established in *A. thaliana* for *ATCOPIA78*, which transposes at high rates following its transient reactivation by heat-stress, but only when RdDM is impaired [136]. Thanks to the development of TE sequence-capture approaches, which enable the massively parallel and highly sensitive detection of transposition events [48], observations first reported for *ATCOPIA78* have now been extended to many other TE families and to at least one additional biotic stress [54]. These new analyses indicate also that for a few additional TE families, impaired RdDM alone is sufficient to induce transposition. Remarkably, the natural hypomorphic variants of RdDM that segregate in *A. thaliana* are likewise associated with higher transposition. Moreover, these alleles are predominantly found in the extreme environments present at the edge of the species niche, where higher transposition rates appear to be positively selected [54]. Taken together, these observations support the notion that TEs, through their unique environmental sensitivity and epigenetic properties, contribute significantly to evolvability, that is, to the ability of organisms to produce heritable phenotypic variation that is adaptive [137].

### (c) Evolutionary significance of TE-associated epiallelic variation

The functional consequences of epiallelic variation have triggered numerous discussions over its evolutionary significance, notably in terms of rapid adaptation in the face of abrupt environmental changes (but see also McGuigan *et al.* [138] in this issue for a discussion of how epigenetics may contribute to adaptation to climate change). It was even suggested that epiallelic inheritance represents the molecular underpinning of Waddington's genetic assimilation [139]. However, it is clear from the evidence discussed above that the adaptive potential provided by stable epiallelic variants in the face of environmental challenges suffers from two major limitations: (i) the rate of spontaneous TE-associated epimutations is not significantly higher than that of SNPs, contrary to what was previously thought, and (ii) the amplitude of phenotypic variation they may cause is relatively small. By contrast, environmentally induced epialleles, which are mainly transient, can arise at once throughout the genome and contribute directly to stress responses. Thus, these two flavours of epiallelic variation (figure 2) should be distinguished as they have distinct evolutionary implications. On the one hand, pronounced and stable epialleles resulting from spontaneous loss of DNA methylation can generate heritable phenotypic variation and can be seen as a form of diversified bet-hedging strategy. On the other hand, moderate and transient epialleles induced by the environment provide a means to generate rapid and transient phenotypic plasticity [140]. However, this second flavour of epimutations appears to be of little adaptive potential in the face of abrupt environmental changes, except perhaps when environments fluctuate. Indeed, modelling suggests that by enabling a rapid loss of stress memory, transient epimutations may be advantageous in the latter context [141–143], and especially when environmental changes are relatively predictable [140,144–147].

An extreme form of transient chromatin-based phenotypic plasticity in response to predictable environmental changes can be found in the vernalization response in *A. thaliana*, which does not involve regulation by DNA methylation. Briefly, accessions vary in their requirement for a cold winter in order to flower in the following spring and this requirement is underpinned by the cold-induced repression of specific alleles of *FLOWERING LOCUS C (FLC),* which encodes a major repressor of flowering [148]. The maintenance of *FLC* silencing past winter relies on trimethylation of lysine 27 of histone H3, which is deposited by polycomb repressive complexes. This prolonged epigenetic silencing is ultimately reversed during reproduction and embryo development [149,150]. Resetting of *FLC* at each generation [151] is essential, as it ensures that the requirement for winter is re-established at each generation. The fact that such reprogramming likely entails a high metabolic cost and is yet clearly adaptive illustrates the evolutionary advantage of preventing the transmission across generations of epivariants induced by seasonal cues.

Although reprogramming of overall DNA methylation is limited in plants, RdDM-dependent CHH methylation is actively removed and re-established during sexual reproduction [98]. Thus it is tempting to draw a parallel with the active resetting of *FLC* expression at each generation and to propose that transgenerational inheritance of DNA methylation variants at TE-containing alleles would in most cases be disadvantageous. Indeed, a few lines of evidence suggest that the transmission of environmentally induced epimutations across multiple generations is not advantageous in habitats where environmental conditions are highly fluctuating [81,130,152], as is the case throughout most of *A. thaliana*'s range. Moreover, models predict that stable epigenetic inheritance is favoured when changes in the environment persist for long periods [153] and is maladaptive otherwise [154]. However, the adaptive potential of heritable environmentally induced epiallelic variation at TE-containing alleles remains to be determined in plants with other life strategies. Extending studies to non-model organisms, notably species relying on asexual propagation or with long perennial vegetative phases, may uncover conditions (e.g. invasions, as reviewed by Mounger *et al.* [101] in this issue) where increased heritability of environmentally induced epiallelic variation is favoured.

## 4. Conclusion and future directions

The experimental demonstration in plants that numerous TE-associated DNA methylation variants can be stably transmitted across several generations as epialleles, i.e. independently of any DNA sequence change, has raised considerable interest in their contribution to the evolutionary process. Here, we have reviewed the molecular studies, mostly in *A. thaliana* but also increasingly in other species, maize in particular, on the types, potential sources, and functional consequences of TE-associated epivariation to reassess its evolutionary significance.

First, it is now evident that TE-associated epivariants come in different flavours, which affect considerably their epiallelic properties. Specifically, stability across generations is often observed when loss of DNA methylation is pronounced and affects cytosines in the three contexts CG, CHG and CHH. By contrast, when only partial, loss of methylation is efficiently corrected by RdDM during sexual reproduction, thus considerably limiting its inheritance.

In nature, genetic modifiers affect RdDM or other DNA methylation pathways only partially, presumably because of the strong deleterious effects of null mutations, and they are, therefore, unlikely to be the main determinants of stable TE-associated epiallelic variation. Nonetheless, the multiplicity of TE-associated epivariants induced by these *trans* modifiers across the genome may contribute collectively to heritable differences in quantitative traits, notably stress responses. However, because of the multiplicity of loci and TE insertion polymorphisms involved, measuring this contribution may be an impossible task for the foreseeable future, thus extending further the unbridgeable gap between complex traits and traditional molecular biology [155].

Likewise, environmentally induced epivariation typically takes the form of moderate DNA methylation changes and it is consistently of limited transgenerational stability in experimental settings. Although this type of epivariation cannot contribute to heritable adaptations because of its transient nature, it is uniquely suited to be invoked at once across the genome and therefore to produce concerted, multigenic expression responses to biotic and abiotic environmental insults.

By contrast to these two sources of moderate epivariation, spontaneous severe epivariation can be stably transmitted across generations, suggesting that it is the prevalent source of heritable TE-associated epialleles in nature. However, their rate of occurrence across the genome is not significantly higher than that of SNPs, which limits their potential to contribute to rapid adaptation.

Irrespective of the origin and stability of TE-associated epivariants, the experimental demonstration of their functional impact at the individual level has been very limited so far. Such endeavours should be greatly facilitated by the newly offered possibilities of targeted epigenome editing using CRISPR-dCas9 systems with methylase or demethylase activity [156,157].

Most of our conclusions need to be tempered by the fact that plant species differ considerably in their TE content, life history and modes of reproduction, which may affect not only the genomic patterning of DNA methylation [105] but also the generation as well as the stability of TE-associated epivariants. For instance, while stable inheritance of environmentally induced epialleles may be deleterious in *A. thaliana*, a fast-cycling annual, it may in fact be advantageous in long-lived perennial species or in species that reproduce asexually. Moreover, most mechanistic insights were derived from studies in *A. thaliana* that considered only the reference accession and the corresponding reference genome. Given the extensive diversity of TE landscapes within this species, it is possible that the epiallelic properties defined in the reference accession may differ substantially between accessions, as discussed above.

Despite these potential differences among plant species, a global picture emerges where natural selection is unlikely to act directly on TE-associated epivariants, but rather on the corresponding TE-containing alleles. Moreover, given their environmental sensitivity, it is tempting to speculate that TE-containing alleles are key determinants of phenotypic plasticity in plants. In turn, they could provide a mechanistic basis for the notion first formulated by C. H. Waddington, and amply confirmed since, that phenotypic plasticity is a genetic property and as such represents a character upon which natural selection can act (see review by Loison [158] in this theme issue for a historical perspective).

**Figure 4.**
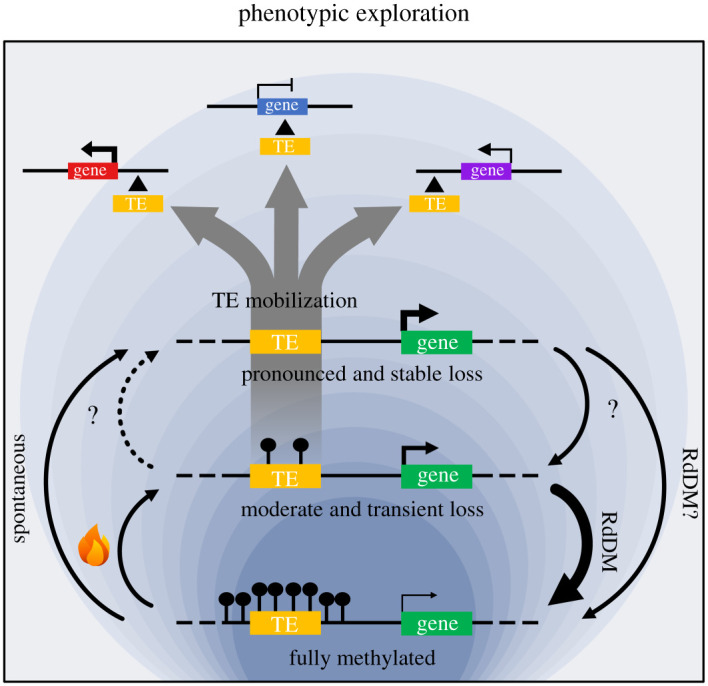
Schematic of the contribution of TE-associated epivariation to the exploration of the phenotypic space via TE mobilization.

The fact that environmentally induced loss of DNA methylation over TEs, even transient, can potentially trigger their mobilization is perhaps the most evolutionarily relevant attribute of TE-associated epivariation. Indeed, while the epiallelic memory of environmental stresses may be lost within one or two generations, its translation into the creation of new TE-containing alleles, often with similar epigenetic properties, provides hard-wired opportunities for the flexible exploration of the phenotypic space (figure 4). Presumably as a result of evolutionary adjustments, some TEs exhibit marked insertion preferences towards environmentally responsive genes [115], thus further enhancing the adaptive value of this exploration. Finally, the central role of RdDM in modulating the epiallelic potential of TEs appears also to be exploited in nature to fine tune the environmental sensitivity of TE mobilization. Together, these considerations and findings highlight how TE-associated epiallelic variation, by its capacity to modulate TE mobilization in response to the environment, endows plant genomes with a powerful engine for rapid adaptation.

## Data accessibility

This article has no additional data.
